# Nonshared environmental factors in the aetiology of autism and other neurodevelopmental conditions: a monozygotic co-twin control study

**DOI:** 10.1186/s13229-022-00487-5

**Published:** 2022-02-19

**Authors:** Johan Isaksson, Vladislav Ruchkin, Nikolas Aho, Karl Lundin Remnélius, Peter B. Marschik, Sven Bölte

**Affiliations:** 1 Child and Adolescent Psychiatry Unit, Department of Medical Sciences, Uppsala University, Uppsala, Sweden; 2 Center of Neurodevelopmental Disorders (KIND), Centre for Psychiatry Research, Department of Women’s and Children’s Health, Karolinska Institutet and Child and Adolescent Psychiatry, Stockholm Health Care Services, Region Stockholm, Stockholm County Council, BUP-FOU Centrum, Gävlegatan 22, 113 30 Stockholm, Sweden; 3 Child Study Center, Yale University School of Medicine, New Haven, CT USA; 4 Child and Adolescent Psychiatry and Psychotherapy, University Medical Center Göttingen and Leibniz ScienceCampus Primate Cognition, Göttingen, Germany; 5 iDN - Interdisciplinary Developmental Neuroscience, Division of Phoniatrics, Medical University of Graz, Graz, Austria; 6 Child and Adolescent Psychiatry, Stockholm Health Care Services, Region Stockholm, Stockholm, Sweden; 7 Curtin Autism Research Group, Curtin School of Allied Health, Curtin University, Perth, WA Austria

**Keywords:** ADHD, Autism, IQ, Early adversities, Non-shared environment, Twins, Risk factors

## Abstract

**Background:**

A significant proportion of variation in likelihood of neurodevelopmental conditions (NDCs) has been attributed to nonshared environmental (NSE) factors, although it remains unclear which NSE factors pose specific risks for certain NDCs.

**Methods:**

A monozygotic co-twin design was applied in a sample of 224 twins (mean age = 17.70 years, SD = 6.28) controlling for confounders such as genes and shared environment. Generalized estimating equation models were fitted, using perinatal and postnatal indications of NSEs as exposure, operationalized both as separate risk factors and as cumulative risk loads. Categorical and dimensional operationalizations of autism spectrum disorder (ASD), attention-deficit/hyperactivity disorder (ADHD), intellectual disability and other NDCs were used as outcomes.

**Results:**

Birth weight discordance was associated with dimensional autism and ADHD for the smaller twin, and medication during infancy was associated with dimensional autism. Among postnatal factors scarlet fever during early childhood was associated with lower IQ. Especially autism was associated with a greater cumulative perinatal or postnatal risk load.

**Limitations:**

When exploring the associations between each condition and specific NSEs the risk of being statistically underpowered increases. Hence, we limit the reported findings on specific indicators of NSEs to trait levels and present descriptive data for categorical NDCs.

**Conclusions:**

The findings support previous research by indicating an association between exposure to perinatal and postnatal risks and subsequent NDCs within twin pairs and suggest that autism may be especially linked to accumulative early environmental risks. The findings are potentially important for developmental outcomes prognoses and may inform targeted prevention and early interventions.

**Supplementary Information:**

The online version contains supplementary material available at 10.1186/s13229-022-00487-5.

## Background

Neurodevelopmental conditions (NDCs) are childhood onset disabilities that persist into adulthood and are associated with altered architecture, maturation, and functioning of the developing central nervous system [1]. NDCs affect a wide range of mental functions, such as attention, executive control, top-down versus bottom-up processing, speech-language and communication, motivation, motor control, learning, and social awareness [2, 3]. In the Diagnostic and Statistical Manual of Mental Disorders 5th ed. (DSM-5) and the International Classification of Diseases 11th Revision (ICD-11), the umbrella concept of NDCs includes attention-deficit/hyperactivity disorder (ADHD), autism spectrum disorder (ASD), intellectual disabilities (ID), communication disorders, specific learning disorders, and motor disorders, such as tics and developmental coordination disorder (DCD) [4, 5]. Most NDCs are viewed as extreme manifestations of traits that are continuously distributed in the general population [6]. Even though different NDCs are associated with similar neurocognitive alterations, with an overlap of symptoms and frequent coexisting psychiatric and somatic problems, they differ with regard to their genetic vulnerabilities, clinical course, prognosis and response to treatment [1]. Therefore, the need for both grouping NDCs together while retaining diagnostic distinctions, as well as for including both categorical diagnoses and dimensional traits, has been emphasised [1].

NDCs are common paediatric challenges, with an estimated prevalence of 17.8% in the years 2015 to 2017 in the USA [7]. Although considered highly heritable, a proportion of NDC causality has been attributed to environmental factors [8], especially *nonshared environmental* (NSE) effects, i.e., exposures unique to different children within the same family that make them differ from each other. For example, twin–twin differences in autistic traits are substantially influenced by NSEs at a clinical severity level [9] and estimates of the NSE impact on ADHD and ASD range between 17 and 44% [10–12]. Importantly, the identification of valid NSEs in the aetiology of NDCs might facilitate research on prevention of early adverse influences on the developing child.

NSE, or rather indicators of NSE, include prenatal factors such as gestational hypertension and diabetes, gestational bleeding, maternal use of medication and teratogens [13–16]; perinatal factors, such as breech presentation, foetal distress, preterm birth, caesarean delivery, birth complications, or birth weight [8, 13, 17]; and postnatal factors such as infections and antibiotic exposure [18, 19]. Despite reported associations between NSE and NDCs, the evidence to support a causal relationship remains insufficient [16] and given the considerable overlap between different types of NDCs [1] it remains to be determined if the associations are generic to NDCs or if certain environmental factors are linked to specific conditions. For instance, the interaction between the same NSEs with different genetic vulnerabilities may result in an elevated likelihood for specific NDCs, or the association between NSEs and NDCs may be across diagnoses/conditions, where changes in brain development may mediate the association by means of shared neurocognitive alterations.

Thus, research designs are needed that control for the broad range of familial factors (genetics and shared environmental factors) indicated in NDCs [17]. The discordant monozygotic (MZ) co-twin design which automatically controls for both genetic factors (MZ twins, also called identical, are assumed to share essentially 100% of each other’s genes) and shared environment (including the prenatal environment) offers unique opportunities to investigate possible causal pathways to NDCs by focusing on the within-twin pair differences with respect to unique environmental experiences [20]. There is however a shortage of studies investigating MZ twin populations that are sufficiently phenotyped for concordance and discordance with regard to NDCs. In addition, most previous twin-studies were limited by small sample size, a lack of standardized assessments, and by overly focusing on single NDC diagnoses. In spite of these limitations, some support has been found for a within pairs association between lower birth weight and ASD traits [21]. Similarly, within pairs, the twin with lower birthweight had a higher risk of having ADHD [22] and traits of ADHD [23]. Birthweight discordance has also been associated with cognitive and verbal developmental delay [24] and lower IQ performance at 3 years of age [25] in the lower weight twin. Twin studies have also reported an association between perinatal hypoxia and respiratory distress and ASD [26] and DCD [27]. In addition, the cumulative load of early medical events has been linked to both ASD and autistic trait severity [28, 29].

In sum, while the general importance of NSE for NDCs is undisputed, it remains unclear which NSE factors may pose a specific risk for a certain NDC and whether exposure to several NSEs may further increase the likelihood of a neuroatypical development. Therefore, this study utilized a MZ co-twin design in order to (1) explore if a wide range of NSE are associated with dimensional (traits) operationalizations of NDCs within twin pairs, (2) test whether different NDCs are associated with different NSE factors and (3) investigate the role of cumulative exposure to several NSE factors for categorical (diagnosis) and dimensional operationalizations of NDCs within twin pairs.

## Materials and methods

### Sample and study design

In this MZ co-twin study, a total of 224 participants (112 MZ same-sex twin pairs) were included (48.2% females; mean age = 17.70 years, SD = 6.28, range: 8–33 years). In total, 49 participants had a diagnosis of ASD (21 females), 43 ADHD (14 females), 13 ID (5 females), 30 other NDCs (e.g., communication disorders, specific learning disorders or motor disorders; 8 females), and 138 typically developed (i.e., with no NDC diagnosis). Thirty-five participants had two or more NDC diagnoses and were included in more than one of the above categories. In total, 52 pairs were concordant for NDCs and 68 pairs were discordant for NDCs (note that concordant pairs may be discordant at the trait level with within-pair differences in dimensional score points). A subsample was included in a previous publication [28] and the present study adds to the previous research by focusing specifically on MZ twins and by including and comparing risk factors for different types of NDCs. The sample was recruited from the Roots of Autism and ADHD Twin Study Sweden (RATSS), an ongoing study that includes twins from the population-based Child and Adolescent Twin Study in Sweden [30] and the Young Adult Twins in Sweden Study, where one or both twins positively screened for ASD or ADHD, but also includes typically developing controls [31]. Included twins are comprehensively clinically phenotyped, and zygosity is determined on a panel of 48 single nucleotide polymorphisms [32]. All background and diagnostic information, including retrospective assessment of NSE risk factors were obtained at the same time, during a diagnostic visit at the clinic and complemented by medical records. The study was approved by the National Swedish Ethical Review Board, and written and oral informed consent was obtained from all participants after the nature of the study procedure had been fully explained.

### Measures

Clinical DSM-5 consensus diagnoses of NDCs were determined by a group of clinicians during the twins’ 2½ day visit at a clinical research unit using medical history records, diagnostic interviews and by first choice standardized diagnostic tools (for more detail and references please see [31, 33]). These include the Schedule for Affective Disorders and Schizophrenia for School-Age Children-Present and Lifetime Version (K-SADS-PL) and the Structured Clinical Interview for DSM-IV-Axis I Disorders (SCID-I), depending on the participant’s age; the Diagnostic Interview for ADHD in adults (DIVA); the Autism Diagnostic Interview—Revised (ADI-R) and the Autism Diagnostic Observation Schedule Second Edition (ADOS-2, modules 3 and 4), neuropsychological tests; and the Adaptive Behavior Assessment System-2 (ABAS-2). When DSM-IV specific instruments were used, a translation to DSM-5 criteria was made by the team of clinicians.

The total raw score points of the parent-report version of the Social Responsiveness Scale Second Edition (SRS-2) were applied to measure autistic traits [34, 35]. The questionnaire was completed during the visit at the clinical research unit. The SRS includes 65 items on interpersonal behavior, communication, inflexible, restricted, and repetitive interests and behaviors. The items are rated on a 4-point Likert-scale ranging from 0 (“not true”) to 3 (“almost always true”), resulting in a total score between 0 and 195, a higher score indicating more autistic traits. Differences of  ≥ 10 raw points have in previous research been deemed as meaningful [34]. For this study, SRS was used as a continuous measure.

ADHD traits were measured during the visit at the clinical research unit with the DSM-oriented ADHD subscale of the parent-rated Child Behavior Checklist (CBCL) and the Adult Behavior Checklist (ABCL) depending on the participants age [36]. The items were rated from 0 (“not true”) to 2 (“very often true”), with higher scores indicating more inattention, hyperactivity and impulsivity. The total score could range between 0 and 14 in the CBCL and 0 and 26 in the ABCL. For this study however, CBCL and ABCL score were separately standardized into a *Z*-score and merged.

The General Ability Index (GAI) of the Wechsler Intelligence Scales for Children or Adults-IV (WISC-IV/WAIS-IV) was used to assess IQ. The GAI is a composite score that is based on three Verbal Comprehension and three Perceptual Reasoning subtests, and the score can range between 40 and 160 where GAI scores between 90 and 109 are considered average.

### Nonshared environmental (NSE) factors

Medical and developmental history with focus on the first 5 years of life was collected from the RATSS parent-report questionnaire completed during the visit at the clinical research unit, designed to cover pre-, peri- and postnatal factors and child medical history. The questionnaire has been validated against medical registry data [28]. Based on 13 MZ pairs discordant for NDCs and 13 MZ TD pairs, information on birthweight showed an excellent agreement with the medical records (ICC = 0.93, CI = 0.88–0.96), whereas the agreement was moderate for cumulative load of early medical events (ICC = 0.55, 95% CI = 0.22–0.75) [28]. Information on agreement for specific indicators of NSEs is shown in more detail in Additional file 1: Table S1. Most questions had a yes/no response format with a request to specify for positive answers. When information on any particular risk factor was left blank (less than 5% of all reports) it was considered as non-present. As specific and separate exposure information for each twin is needed for an informative within-pair analysis, which is often impossible to derive in twins that share prenatal milieu, the analyses of prenatal environmental factors were not included in this study. For the analyses of perinatal and postnatal factors, we selected those which have been highlighted in previous research on psychiatric and NDCs [8, 13, 17–19].

### Perinatal factors

*Birthweight* information for each twin was collected with a low birthweight defined as ≤ 2000 g (scored as 1), a definition applied in previous twin studies, e.g., [37]. In addition, considering that twin weight discordance is an indicator of divergent twin growth and that the difference among the twins’ birthweight at 15–25% is associated with an increased risk of morbidity and mortality rates as well as other perinatal complications [38], the *twin birth weight discordance* was used as an additional risk factor. The cut-off for the birth weight discordance ([larger twin weight − smaller twin weight]/larger twin weight × 100) indicating the potential risk was set at 18% or greater, using a stringent approach suggested by Breathnach et al. [39], where the twin with a lower birthweight was coded as 1. Parent-reported *convulsions, oxygen therapy* (as an indicator for hypoxia and respiratory distress)*, phototherapy* (as an indicator for hyperbilirubinemia)*, breech presentation,* and *use of medication* (e.g., antibiotics, iron) during infancy, were also coded as 1 if present. A cumulative risk load score was calculated as a sum of the single risk factors, with a possible range from 0 to 7, a higher score indicating more adverse perinatal factors.

### Postnatal factors

Parents’ reports on whether their child had experienced *measles, jaundice, mumps, scarlet fever, asthma, frequent ear infections, head injury, convulsions* or *heart disease* during early childhood were collected, and a cumulative load was generated based on the single events ranging between 0 and 9, with a higher score indicating more postnatal risk.

### Statistical analyses

**Fig 1:**
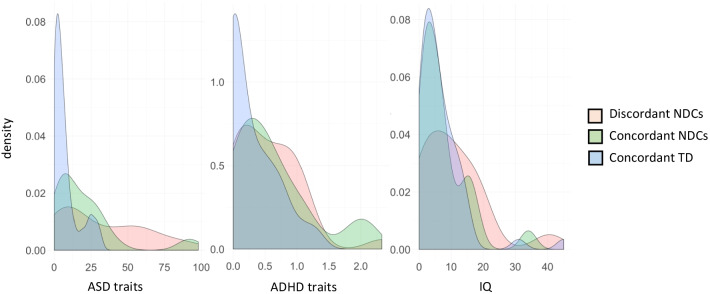
Within-pair difference in the distributions on autistic traits, traits of ADHD and IQ by concordance for neurodevelopmental conditions (NDCs)

Within-pair distributions of autistic traits, traits of ADHD and IQ in terms of the level of twin-twin discordance are shown in Fig. 1. We examined within-pair association between NSE factors and NDCs using conditional regression analyses within the generalized estimating equations (GEE) framework implemented in the “drgee” package of R which does not make any distributional assumptions [40, 41]. In this co-twin design, the difference in the exposure variable within a pair is correlated to the difference in the outcome variable within the same pair (see Additional file 2: Fig. S1). For the continuous outcome (i.e., traits and number of NDCs), we used the identity link within the drgee function, and for binary outcomes (i.e., diagnosis of NDC) we used the log link within the drgee function and the log risk ratio (RR) are presented in back transformed format. The framework accounts for the use of related individuals in the analysis, including calculation of robust standard errors [40, 42]. In the regressions, all factors shared within clusters (here: twin pairs) are adjusted for. In the case of twins, these include additive and non-additive genetic variants, and environmental exposures that increase familial resemblance (i.e., shared environment). No other factors were adjusted for in the models. Two models were fitted for peri- and postnatal NSEs separately. First, we conducted a set of analyses with the single peri- and postnatal NSEs as exposures and dimensional NDCs as separate outcomes. Since the model reduces the number of informative participants for using categorical outcomes (being discordant for specific NDC and also being discordant for exposure), we only present descriptive data for diagnostic outcome. Second, the cumulative environmental risk loads for perinatal and postnatal factors were used as exposure and number of NDC diagnoses, specific NDCs, i.e., ASD, ADHD, ID, as well as autistic traits, traits of ADHD and IQ, were used as separate outcomes. In addition, all other NDCs were combined into one category. For the cumulative environmental risk loads, all included NSEs were weighted the same (i.e., present or not-present). *P*-values < 0.05 were considered significant. The Benjamini–Hochberg false discovery rate correction (FDR corr) of 0.05 was additionally applied in order to correct for multiple comparisons [43]. We also present confidence intervals and *Z* statistics for the reported associations.

## Results

Figure 1 depicts the distribution of and differences in the key dimensional constructs in terms of level of twin-twin discordance. In nine pairs twin-to-twin transfusion was reported. One participant had reported hydrocephalus, one cerebral haemorrhage, one craniosynostosis, one encephalitis and one had meningitis during childhood. Among the participants, 27.7% of the twins had one, 12.9% two and 30.9% three or more perinatal risk factors reported. The corresponding numbers for postnatal risk factors were 32.1% for one, 16.1% for two and 7.1% for three or more cumulative factors. Partial correlation, adjusting for sex and age, between the NSEs and NDCs are presented in Additional file 3: Table S2.

### Non-shared perinatal factors

Table 1 shows the percentage of twins with single perinatal risk factor exposure and mean value of the cumulative risk load stratified by categorical NDC concordance, while Table 2 shows the models for neurodevelopmental trait outcomes. Analyses showed that in pairs with birth weight discordance ≥ 18%, the lower weighted twin has a higher risk of autistic trait score and higher traits of ADHD. More specifically the lower weighted twin scored almost half a standard deviation higher on the SRS-2 (13.9 raw points) and the CBCL/ABCL ADHD subscale compared to their co-twin. The association between birth weight discordance and traits of ADHD, but not ASD traits, remained when applying FDR corr. As shown in Table 1, all of those reporting ≥ 18% lower birth weight in discordant pairs had either a diagnosis of ASD or ADHD. The association between birth weight discordance and autistic traits and traits of ADHD, as well as lower IQ, for the smaller twin was also present when applying birthweight as a continuous measure, see Additional file 4: Table S3. The twin who received medication during infancy had higher levels of autistic traits compared to their co-twin, a finding that remained after FDR corr.

**Table 1:** Number of reported perinatal and postnatal risk factors as exposures stratified by neurodevelopmental disorders and concordance

	ASD		ADHD		Other NDCs		ID		TD
	Concordant 13 pairs^a^	Discordant 23 pairs^b^ affected/unaffected	Concordant 14 pairs	Discordant 15 pairs affected/unaffected	Concordant 8 pairs	Discordant 14 pairs affected/unaffected	Concordant 3 pairs	Discordant 7 pairs affected/unaffected	Concordant 51 pairs
*Perinatal risks*									
Perinatal risk load Mean (SD)	1.46 (1.65)	2.22 (1.93)/1.48 (1.53)	1.00 (1.52)	2.13 (2.10)/1.73 (1.79)	0.75 (1.24)	1.79 (1.63)/1.36 (1.55)	1.83 (1.72)	2.86 (2.48), 2.57 (2.37)	1.10 (1.20)
Birth weight ≤ 2000 g (*n* = 51)	23.1%	47.8/39.1%	10.7%	28.6/28.6%	12.5%	35.7/28.6%	0%	57.1/57.1%	11.8%
Growth discordance ≥ 18% (*n* = 28)	7.7%	26.1/0%	7.1%	28.6/0%	0%	28.6/14.4%	0%	42.9/14.3%	10.8%
Convulsions (*n* = 9)	11.5%	13.0/4.3%	7.1%	14.3/14.3%	0%	0/0%	33.3%	28.6/14.3%	2%
Oxygen therapy (*n* = 63)	42.3%	34.8/30.4%	17.9%	50.0/28.6%	12.5%	28.6/42.9%	50.0%	57.1/57.1%	20.6%
Phototherapy (*n* = 91)	38.5%	52.2/47.8%	28.6%	50.0/50.0%	37.5%	42.9/35.7%	50.0%	57.1/57.1%	38.2%
Breech position (*n* = 36)	7.7%	30.4/17.4%	14.3%	28.6/35.7%	0%	28.6/0%	16.7%	0/14.3%	15.7%
Medication (*n* = 26)	15.4%	17.4/8.7%	14.3%	21.4/21.4%	12.5%	14.3/14.3%	33.3%	42.9/42.9%	5.9%
*Postnatal risks*									
Postnatal risk load Mean (SD)	0.88 (1.03)	1.61 (1.37)/1.09 (1.24)	1.00 (0.98)	1.20 (1.70)/0.87 (1.30)	1.25 (1.00)	0.64 (1.08)/ 0.50 (0.85)	2.50 (1.05)	1.71 (2.29), 1.29 (1.60)	0.72 (0.83)
Measles (*n* = 2)	0%	0/0%	0%	0/0%	0%	0/0%	0%	0/0%	2%
Jaundice (*n* = 20)	19.2%	21.7/21.7%	0%	14.3/21.4%	7.1%	7.1/7.1%	33.3%	28.6/28.6%	13.7%
Mumps (*n* = 2)	0%	0/4.3%	0%	0%	0%	0/7.1%	0%	0/0%	0%
Scarlet fever (*n* = 20)	19.2%	13.0/13.0%	17.9%	14.3/14.3%	12.5%	7.1/14.3%	33.3%	14.3/14.3%	4.9%
Asthma (*n* = 47)	11.5%	30.4/30.4%	21.4%	35.7/28.6%	50.0%	0/7.1%	33.3%	28.6/14.3%	15.7%
Frequent ear infections (*n* = 50)	15.4%	43.5/26.1%	28.6%	14.3/7.1%	37.5%	28.6/14.3%	66.7%	42.9/42.9%	17.6%
Head injury (*n* = 27)	3.8%	21.7/8.7%	17.9%	14.3/14.3%	18.8%	14.3/0%	16.7%	14.3/14.3%	13.7%
Convulsions (*n* = 9)	11.5%	17.4/0%	10.7%	21.4/0%	6.3%	0/0%	33.3%	28.6/0%	1.0%
Heart disease (*n* = 7)	7.7%	13.0/4.3%	3.6%	14.3/7.1%	0%	7.1/0%	33.3%	14.3/14.3%	1.0%

ADHD = attention-deficit/hyperactivity disorder, ASD = autism spectrum disorder, CI = confidence interval, ID = intellectual disability, NDCs = neurodevelopmental conditions (other NDCs includes e.g., communication disorders, specific learning disorders or motor disorders), TD = typically developing

^a^13 pairs = 26 individuals, ^b^23 discordant pairs = 23 affected twins where their co-twin (i.e., 23 individuals) is not affected

**Table 2:** Results from within-pair linear regression analyses with perinatal and postnatal risk factors as main predictors of trait Neurodevelopmental conditions

	ASD traits^a^ *b* (95% CI), *p*-value, *Z*-score	ADHD traits^b^ *b* (95% CI), *p*-value, *Z*-score	IQ^c^ *b* (95% CI), *p*-value, *Z*-score
*Perinatal risk load*	8.30 (3.92, 12.67), 0.0002*, 3.72	0.10 (− 0.01, 0.22), 0.083, 1.73	− 1.43 (− 3.29, 0.43), 0.131, − 1.51
Birth weight ≤ 2000 g	8.46 (− 6.53, 23.44), 0.269, 1.11	0.22 (− 0.11, 0.55), 0.198, 1.29	− 1.55 (− 9.44, 6.35), 0.701, − 0.38
Birth weight discordance ≥ 18%	13.89 (4.22, 23.57), **0.005**, 2.81	0.41 (0.16, 0.66), 0.001*, 3.26	− 3.44 (− 8.69, 1.81), 0.199, − 1.29
Convulsions	18.00 (− 3.36, 39.36), 0.099, 1.65	0.05 (− 1.16, 1.25), 0.940, 0.08	− 9.60 (− 24.11, 4.91), 0.195, − 1.30
Oxygen therapy	11.47 (− 4.88, 28.81), 0.169, 1.38	0.13 (− 0.16, 0.42), 0.379, 0.88	− 1.43 (− 6.58, 3.72), 0.587, − 0.54
Light treatment	− 1.23 (− 8.16, 5.70), 0.728, − 0.35	− 0.18 (− 0.54, 0.19), 0.352, − 0.93	− 3.33 (− 9.47, 2.80), 0.287, − 1.07
Breech position	10.20 (− 3.24, 23.64), 0.137, 1.49	0.10 (− 0.22, 0.41), 0.552, 0.59	1.27 (− 3.95, 6.49), 0.633, 0.48
Medication	21.25 (8.29, 34.12), 0.001*, 3.21	− 0.29 (− 0.63, 0.50), 0.095, − 1.67	− 1.75 (− 9.07, 5.67), 0.639, − 0.47
*Postnatal risk load*	9.48 (3.23, 15.66), 0.0029*, 2.98	0.08 (− 0.11, 0.27), 0.399, 0.84	− 2.97 (− 5.67, − 0.28), **0.031**, − 2.16
Measles	–	–	–
Jaundice	6.54 (− 5.70, 18.79), 0.295, 1.05	− 0.10 (− 0.60, 0.40), 0.700, − 0.39	− 6.73 (− 11.97, − 1.48), **0.012**, − 2.52
Mumps	− 33.50 (− 70.40, 3.40), 0.075, − 1.78	− 0.21 (− 1.22, 0.80), 0.683, − 0.41	− 11.00 (− 24.93, 2.92), 0.121, − 1.55
Scarlet fever	3.25 (− 9.46, 15.96), 0.616, 0.50	− 0.14 (− 0.82, 0.54), 0.688, − 0.40	− 5.25 (− 6.70, − 3.79), < 0.0001*, − 7.01
Asthma	− 7.38 (− 24.75, 10.00), 0.406, − 0.83	0.15 (− 0.24, 0.53), 0.455, 0.75	3.12 (− 4.22, 10.46), 0.405, 0.83
Frequent ear infections	12.83 (0.67, 24.99), **0.039**, 2.07	0.02 (− 0.31, 0.35), 0.900, 0.13	2.17 (− 8.90, 4.57), 0.528, − 0.63
Head injury	14.84 (0.16, 29.53) **0.048**, 1.98	0.24 (0.01, 0.47), 0.387, 2.07	− 3.21 (− 8.43, 2.01), 0.228, − 1.21
Convulsions	32.00 (9.29, 54.71), **0.006**, 2.76	− 0.00 (− 0.92, 0.92), 1.000, − 0.00	− 8.33 (− 22.54, 5.87), 0.250, − 1.15
Heart disease	31.60 (− 1.00, 64.20), 0.057, 1.90	0.14 (− 0.15, 0.42), 0.350, 0.93	− 5.50 (− 13.17, 2.17), 0.160, 1.41

– Too few discordant pairs for calculating estimates

The results were obtained from separate models

Bold indicate *p* < 0.05 uncorrected

^*^Significant result after correcting for multiple comparisons using Benjamini–Hochberg procedure with false discovery rate (FDR) set at 5%

ADHD = attention-deficit/hyperactivity disorder, ASD = autism spectrum disorder, CI = confidence Interval

^a^Measured with Social Responsiveness Scale-2 (SRS-2)

^b^Measured with the Child Behavior Checklist (CBCL) or the Adult Behavior Checklist (ABCL)

^c^Measured with Wechsler Intelligence Scales for Children or Adults-IV (WISC-IV/WAIS-IV)

Tables 2 and 3 show the models for dimensional and categorical neurodevelopmental outcomes with cumulative loads of NSE risk factors as exposure. Cumulative load of perinatal factors was associated with an increase in number of NDC diagnoses. When investigating specific NDCs, the perinatal risk load contributed to ASD and autistic traits but not to any other traits or NDC diagnoses. After applying the FDR corr, the association between the perinatal risk load and increase number of NDC diagnoses and autistic traits remained.

**Table 3:** Results from within-pair analyses with perinatal and postnatal risk loads as main predictors of categorical Neurodevelopmental conditions

	nr of NDC diagnoses *b* (95% CI), *p*-value, *Z*-score	ASD diagnosis *RR* (95% CI), *p*-value, *Z*-score	ADHD diagnosis *RR* (95% CI), *p*-value, *Z*-score	Other NDCs *RR* (95% CI), *p*-value, *Z*-score	ID *RR* (95% CI), *p*-value, *Z*-score
Perinatal risk load	0.22 (0.10, 0.34), 0.00038*, 3.55	1.78 (1.11, 2.84), **0.016**, 2.41	1.38 (0.96, 1.99), 0.079, 1.76	2.25 (0.52, 9.72), 0.277, 1.09	1.94 (0.46, 8.13), 0.367, 0.90
Postnatal risk load	0.29 (0.05, 0.52), **0.019**, 2.35	1.94 (1.11, 3.40), **0.020**, 2.32	1.33 (0.81, 2.18), 0.264, 1.12	1.29 (0.60, 2.79), 0.513, 0.65	1.45 (1.03, 2.05), **0.035**, 2.11

The results were obtained in separate models. For continuous outcomes linear regression was used, and for binary outcomes logistic risk ratios (RR) were used.

Bold indicates *p* < 0.05 uncorrected

ADHD = attention-deficit/hyperactivity disorder, ASD = autism spectrum disorder, CI = confidence Interval, ID = intellectual disability, NDCs = neurodevelopmental conditions (other NDCs includes e.g., communication disorders, specific learning disorders or motor disorders)

^*^Significant result after correcting for multiple comparisons using Benjamin-Hochberg procedure with false discovery rate (FDR) set at 5%

### Nonshared postnatal factors

As shown in Table 2, postnatal convulsions, frequent ear infections and head injury were associated with autistic traits, but the association did not survive FDR corr. The twin with reported convulsions scored on average 1 standard deviation higher on the SRS-2 (32 raw points) than their co-twin. In addition, scarlet fever and jaundice were associated with lower IQ, and the association between scarlet fever and IQ remained after FDR corr. As shown in Table 1, all of those reporting convulsions in discordant pairs had either diagnosis of ASD, ADHD or ID.

The postnatal cumulative risk load was associated with an increasing number of NDC diagnoses. The cumulative load also contributed to both ASD and autistic traits, as well as ID and IQ, see Tables 2 and 3. After applying the FDR corr, the association between the postnatal cumulative risk load and autistic traits remained.

## Discussion

We investigated the significance of nonshared peri- and postnatal environmental factors in neurodevelopmental conditions in a monozygotic co-twin design. Cumulative perinatal and postnatal risks were associated with neurodevelopmental conditions, and especially autistic traits, within the pairs. For perinatal NSE, divergent twin weight was associated with traits of ASD and ADHD for the smaller twin, and among discordant pairs, only the twin with ASD or ADHD diagnosis had a birthweight ≥ 18% compared to their co-twin. In addition, the twin who received medication during infancy had more traits of ASD compared to their co-twin. For postnatal NSE, the twin with convulsions had more ASD traits, and at a diagnostic level only those with a diagnosis of ASD, ADHD or ID reported convulsions within discordant pairs. In addition, frequent ear infections and head injury were associated with ASD traits, whereas scarlet fever and jaundice were associated with lower IQ.

Since unmeasured familial confounders, such as genes and shared environment are adjusted for, the discordant MZ co-twin design offers unique opportunities to investigate possible causal NSE pathways to NDCs. In our study, twin birth weight discordance was the perinatal factor contributing most to the likelihood of emerging autistic and ADHD traits, as well as lower IQ when applying birthweight as a continuous measure, for the twin with less weight. In line with this, both ASD traits [21], ADHD [22], traits of ADHD [23] and IQ [25] have been previously associated with birth-weight difference, with the smaller twin showing increased severity of problems. Moreover, birth weight discordance may double the risk of other adverse perinatal outcomes among twins, including mortality, hypoxic-ischemic encephalopathy, respiratory distress, or sepsis [39].

Since twins have the same gestational age, a difference in their birth weights occurs due to the factors affecting the individual growth of each twin. Possible mechanisms for discordant growth include twin–twin transfusion syndrome or unequal placental sharing [39], although the association between weight discordance and adverse outcomes in the study by Breathnach and colleagues [39] remained even after excluding twins with transfusion syndrome [37]. Other factors that may influence birth weight discordance include maternal factors, such as older age and tobacco use, fetal risk factors, such as viral infections, and placental factors, such as velamentous cord insertion [38]. Hypothetically, a growth discordance might influence brain development in ways specific to ASD and ADHD, as it has been reported that variation in birth weight among MZ twins may be associated with alterations of brain anatomy persisting into late adolescence with lower birth weight being associated with reduced brain volume, and more specifically with altered cortical surface area [44], a cerebral area of significance to ASD [45]. The use of medication reported in our study, such as antibiotics, during infancy for the twin with higher autistic traits may increase microbiome depletion and bacterial dysbiosis, also reported in children with ASD [18].

For postnatal NSE factors, convulsions were associated with ASD traits, albeit no longer significant when correcting for multiple comparisons using Benjamini–Hochberg FDR corr. Even though the cause of convulsions was not investigated in this study, and only few participants had reported convulsions, previous research suggested that children with epileptic and non-epileptic convulsions in early childhood have a higher degree of neurodevelopmental and psychopathology scores, as compared to controls, at least nine years later [46, 47]. Epilepsy has also been more commonly reported among individuals with ASD, as compared to peers with ADHD [48]. A history of convulsions has also been linked to structural correlates, such as decreased hippocampal volume and white matter tract changes [46]. In our study, head injury during early childhood and frequent ear infections, was associated with more autistic traits, and scarlet fever and jaundice were associated with lower IQ. The association between scarlet fever and IQ remained significant even after using the FDR corr. Even though streptococcal infection has been associated with a wider range of NDCs [49] and exposure to infections in early childhood has been associated with lower IQ in adolescence [50], more research is needed to corroborate our findings. The association with head injury needs to be interpreted with caution because of the moderate degree of agreement between parental reports and medical records and the association lost its significance after the FDR corr.

Generally, our findings are consistent with the previous research on NSE in NDCs [8], indicating, an association between autism and perinatal and postnatal risks within twin pairs. Our finding thus suggests that autism may be particularly sensitive to cumulative early environmental risk load. Previous research has also reported a potential cumulative effect of the number of environmental adversities on the severity of ASD and autistic traits [28, 29, 51]. Accordingly, it has been suggested that ASD may be triggered by additive or cascading effects of high-level exposure to endogenous and environmental factors, hypothesised to affect the immune system and central nervous system functions during critical developmental stages [52]. In contrast with findings from previous twin and sibling studies [8], perinatal hypoxia and respiratory distress were not associated with neurodevelopmental outcomes in our study. This negative finding could reflect limited power in our model and that we used a proxy (oxygen therapy) for respiratory distress. We did however observe 11-raw score points more on the SRS-2 for the twin who received oxygen, although not significant.

## Limitations

In order to fully evaluate the validity of our findings, some limitations of the current study need to be discussed. First, data on NSE were based on retrospective parental inquiry, and may be subject to confirmation or recall bias. To maximize data validity, the self-reported medical information from home-stored medical reports and record-keepings was validated against medical registry data. While ratings on birthweight had an excellent agreement with the medical records, other NSEs had a lower agreement, e.g., good agreement for oxygen therapy (hypoxia and respiratory distress) and moderate agreement for breech position, which should be considered when interpreting the results. Second, the NSE factors should be understood as rather indirect indicators of environmental risks for NDCs, e.g., phototherapy, why future studies should seek to assess primary medical events as authentically as possible, such as hyperbilirubinemia. Third, several other potential indicators of NSEs that may be of importance, including Apgar score, were not included. Since twins share prenatal milieu, prenatal environmental factors such as maternal use of medication and teratogens could not be included in the within-pair analysis. Fourth, considering the design of the study, we were unable to draw any firm conclusions about the possible causality. Even though the MZ-twin design provides a unique opportunity to untangle the contribution of NSEs on development, reverse causality could not be excluded where NDCs or other factors related to these conditions may increase the risk for NSEs in early development. Fifth, although an MZ co-twin design implies a high degree of genetic and shared environment control, there may be post-twining de novo mutations that may contribute to a minority of cases of discordance. Sixth, concerns have been raised that findings in twin samples may not be generalizable to non-twin populations. However, previous research suggests that twins do not systematically differ from the general population of non-twins on measures of behavior and development [53, 54], including the prevalence of autism [55]. Our sample is also biased toward twins discordant for NDCs. Although this recruitment strategy makes it inappropriate to use bivariate twin models such as A (additive genetics) C (common/shared environment) and E (unique environment) ACE models, it is appropriate for investigating the association between environmental risk factors and outcomes while controlling for familial confounders. Seventh, when exploring the associations between each condition and specific indicators for NSEs the risk of being statistically underpowered increases. In addition, as the case with birth weight discordance and postnatal convulsions, no NSE was reported among the non-affected twins, why it is not feasible to obtain confidence intervals for the RRs in a small sample size using standard measures. Therefore, we only include descriptive data on the association between specific NSEs and NDCs. Lastly, the validity of applying a specific *p*-value could be discussed. In this study we conducted numerous statistical calculations which increases the risk for type I errors. At the same time, correction for multiple testing with e.g., Bonferroni correction, has been criticized since the interpretations depend on the number of tests that are performed and the likelihood of type II errors is also increased [56]. In this study, we therefore also present results after correcting for multiple comparisons using Benjamini–Hochberg procedure with FDR corr [43], which are deemed to be less conservative than the Bonferroni correction using familywise error rate.

On balance, the strengths of the study include a relatively large and rare sample of well phenotyped MZ twins, including a considerable number of MZ twins in Sweden concordant and discordant for NDCs (for a specification of number of concordant and discordant pairs from a nation-wide population-based study in Sweden see [57], for a more comprehensive review on previous twin research in ASD see [58]). Furthermore, the applied discordant MZ-twin design is a powerful method for investigating the contribution of NSE to neurodevelopmental conditions.

## Conclusions

The current study offers insights into early risk factors associated with neurodevelopmental problems and provides additional perspectives on prognosis and prevention. Weight discordance at birth was an important risk factor for both autistic and ADHD traits. Medication early in life, as well the presence of convulsions and streptococcal infection in the first year of life were also associated with traits of NDCs in the within-pair analyses. Even though our finding needs to be interpreted with caution due to a small number of pairs being discordant for specific NSEs, the findings extend previous research by applying an MZ co-twin control design with a high degree of genetic and shared environment control. Our results support a relationship between early NSE and NDCs, where autism seems to be particularly sensitive to a cumulative impact of negative early environmental events. As previously suggested [9] and as supported by the present study, autistic traits tend to be continuously distributed even in discordant twins. It may be hypothesized that when the traits approximate a diagnostic threshold, multiple exposures to specific risk factors, such as those described in the present study may act as triggers and aggravate the phenotype to a clinical level. The findings highlight the need for interventions targeting factors contributing to fetal growth reduction including viral infections and maternal health problems. Improving prenatal care might influence a number of risk NSEs. Further identification of valid NSEs in the aetiology of NDCs may facilitate prevention efforts with regard to early adverse influences on the developing child.

## Supplementary Information

**Additional file 1: Table S1.** Results of the intraclass correlation coefficient based on some of the included measurements, consistency agreement 2-way mixed effects model.

**Additional file 2: Fig. S1.** Within-pair association between autistic traits and perinatal or postnatal risk factors in two example pairs.

**Additional file 3: Table S2.** Partial Correlation Coefficients between the study variables, adjusted for age and sex.

**Additional file 4: Table S3.** Results from within-pair linear regression analyses with birthweight difference in gram as main predictors of trait Neurodevelopmental conditions.

## Data Availability

The datasets presented in this article are not readily available because of the regulations in the ethical approval and university policies, requiring among others a data sharing agreement. Requests to access the datasets should be directed to SB, sven.bolte@ki.se.

## Abbreviations

ABAS: Adaptive behavior assessment system
ABCL: Adult Behavior Checklist
ADHD: Attention-deficit/hyperactivity disorder
ADI-R: Autism Diagnostic Inventory-Revised
ADOS: Autism Diagnostic Observation Schedule—Second Edition
ASD: Autism Spectrum Disorder
CBCL: Child Behavior Checklist
DCD: Developmental coordination disorder
DIVA: Diagnostic Interview for ADHD in Adults
DSM: Diagnostic and statistical manual of mental disorders
GAI: General Ability Index
GEE: Generalized estimating equations
ICC: Intraclass correlation coefficient
ICD: International classification of diseases
ID: Intellectual disabilities
IQ: Intelligence quotient
K-SADS-PL: Schedule for affective disorders and schizophrenia for school-age children-present and lifetime version
MZ: Monozygotic
NDCs: Neurodevelopmental conditions
NSE: Nonshared environmental
RATSS: Roots of autism and ADHD Twin Study Sweden
SCID-I: Structured clinical interview for DSM-IV-Axis I disorders
SRS: Social Responsiveness Scale
TD: Typically developing
WISC-IV/WAIS-IV: Wechsler Intelligence Scales for Children or Adults-IV
