# Supplementary material for: Nonshared environmental factors in the aetiology of autism and other neurodevelopmental conditions: a monozygotic co-twin control study
Source: Mol Autism. 2022 Feb 19;13:8. doi: 10.1186/s13229-022-00487-5 (PMC8858556; doi:10.1186/s13229-022-00487-5)
Supplement: Supplementary file 1 — Additional file 1: Table S1. Results of the intraclass correlation coefficient based on some of the included measurements, consistency agreement 2-way mixed effects model. [file 13229_2022_487_MOESM1_ESM.docx]

|  | Medical records versus RATSS questionnaire | |
| --- | --- | --- |
|  | ICC | 95%CI |
| Birth weight | 0.930 | 0.880–0.960 |
| Oxygen therapy | 0.870 | 0.800–0.920 |
| Light treatment (hyperbilirubinemia) | 0.860 | 0.780–0.910 |
| Breech position | 0.550 | 0.280–0.720 |
| Jaundice | 0.655 | 0.392–0.804 |
| Asthma | 0.815 | 0.676–0.894 |
| Frequent ear infections | 0.865 | 0.765–0.923 |
| Head injury | -0.065 | -0.877–0.396 |
| Convulsions (epilepsy) | 0.793 | 0.633–0.883 |
| Heart disease | 0.564 | 0.235–0.751 |

**Supplementary Table 1** Results of the intraclass correlation coefficient based on some of the included measurements, consistency agreement 2-way mixed effects model

ICC = Intraclass Correlation Coefficient, CI = confidence Interval, RATSS = Roots of Autism and ADHD Twin Study Sweden
