# Supplementary material for: Nonshared environmental factors in the aetiology of autism and other neurodevelopmental conditions: a monozygotic co-twin control study
Source: Mol Autism. 2022 Feb 19;13:8. doi: 10.1186/s13229-022-00487-5 (PMC8858556; doi:10.1186/s13229-022-00487-5)
Supplement: Supplementary file 2 — Additional file 2: Fig. S1. Within-pair association between autistic traits and perinatal or postnatal risk factors in two example pairs. [file 13229_2022_487_MOESM2_ESM.docx]

**Supplementary Figure 1** Within-pair association between autistic traits and perinatal or postnatal risk factors in two example pairs

High autistic trait co-twin

High autistic trait co-twin

Low autistic trait twin

Low autistic trait twin

Illustrating the within-pair association between nonshared environmental (NSE) risk factors and autistic traits in two twin pairs (pair one coloured red and pair two blue)
