# Supplementary material for: Nonshared environmental factors in the aetiology of autism and other neurodevelopmental conditions: a monozygotic co-twin control study
Source: Mol Autism. 2022 Feb 19;13:8. doi: 10.1186/s13229-022-00487-5 (PMC8858556; doi:10.1186/s13229-022-00487-5)
Supplement: Supplementary file 3 — Additional file 3: Table S2. Partial Correlation Coefficients between the study variables, adjusted for age and sex. [file 13229_2022_487_MOESM3_ESM.docx]

**Supplementary Table 2** Partial Correlation Coefficients between the study variables, adjusted for age and sex

|  | ASD  Partial *r*  *p*-value | ASD traits^a^  Partial *r*  *p*-value | ADHD  Partial *r*  *p*-value | ADHD traits^b^  Partial *r* *p*-value | ID  Partial *r*  *p*-value | IQ^c^  Partial *r*  *p*-value | Other NDCs  Partial *r*  *p*-value |
| --- | --- | --- | --- | --- | --- | --- | --- |
| **Perinatal risk load** | 0.143 | 0.085 | -0.017 | 0.108 | 0.159 | -0.181 | -0.050 |
|  | **0.034** | 0.210 | 0.796 | 0.115 | **0.017** | **0.007** | 0.455 |
| Birth weight  ≤2000 g | 0.132 | 0.034 | -0.100 | 0.045 | 0.036 | -0.081 | 0.001 |
|  | **0.049** | 0.618 | 0.138 | 0.510 | 0.595 | 0.234 | 0.986 |
| Growth discordance ≥18% | 0.072 | 0.042 | 0.031 | 0.074 | 0.085 | -0.095 | 0.014 |
|  | 0.287 | 0.540 | 0.642 | 0.279 | 0.206 | 0.162 | 0.838 |
| Convulsions | 0.204 | 0.157 | 0.112 | 0.113 | 0.330 | -0.198 | -0.094 |
|  | **0.002** | **0.020** | 0.097 | 0.100 | **<0.001** | **0.003** | 0.162 |
| Oxygen therapy | 0.109 | 0.093 | -0.027 | 0.096 | 0.132 | -0.099 | -0.089 |
|  | 0.104 | 0.170 | 0.686 | 0.161 | **0.049** | 0.144 | 0.188 |
| Light treatment | 0.022 | -0.054 | -0.064 | -0.047 | 0.054 | -0.089 | -0.020 |
|  | 0.739 | 0.422 | 0.342 | 0.494 | 0.427 | 0.188 | 0.766 |
| Breech position | 0.039 | 0.060 | 0.042 | 0.103 | -0.054 | 0.019 | -0.025 |
|  | 0.565 | 0.373 | 0.532 | 0.132 | 0.423 | 0.781 | 0.713 |
| Medication | 0.055 | 0.093 | 0.036 | 0.118 | 0.196 | -0.258 | -0.007 |
|  | 0.414 | 0.171 | 0.591 | 0.085 | **0.003** | **<0.001** | 0.913 |
| **Perinatal risk load** | 0.162 | 0.246 | 0.060 | 0.087 | 0.285 | -0.150 | 0.011 |
|  | **0.016** | **<0.001** | 0.375 | 0.204 | **<0.001** | **0.026** | 0.870 |
| Measles | -0.060 | -0.111 | -0.049 | -0.104 | -0.027 | -0.014 | -0.030 |
|  | 0.372 | 0.100 | 0.468 | 0.130 | 0.687 | 0.841 | 0.658 |
| Jaundice | 0.084 | 0.080 | -0.154 | -0.026 | 0.113 | -0.110 | -0.128 |
|  | 0.212 | 0.234 | **0.022** | 0.711 | 0.093 | 0.105 | 0.057 |
| Mumps | -0.045 | -0.065 | -0.049 | -0.042 | -0.021 | 0.073 | -0.047 |
|  | 0.505 | 0.337 | 0.465 | 0.540 | 0.751 | 0.284 | 0.486 |
| Scarlet fever | 0.108 | 0.213 | 0.082 | 0.211 | 0.104 | -0.093 | -0.022 |
|  | 0.109 | **0.001** | 0.224 | **0.002** | 0.122 | 0.169 | 0.744 |
| Asthma | -0.037 | 0.046 | 0.021 | 0.131 | 0.043 | 0.016 | 0.034 |
|  | 0.586 | 0.494 | 0.754 | 0.055 | 0.519 | 0.817 | 0.615 |
| Frequent ear infections | 0.085 | 0.122 | 0.002 | -0.116 | 0.191 | -0.081 | 0.091 |
|  | 0.209 | 0.072 | 0.975 | 0.090 | **0.004** | 0.234 | 0.178 |
| Head injury | 0.007 | 0.027 | 0.071 | -0.031 | 0.028 | -0.010 | 0.059 |
|  | 0.914 | 0.686 | 0.295 | 0.650 | 0.678 | 0.881 | 0.378 |
| Convulsions | 0.274 | 0.264 | 0.249 | 0.238 | 0.336 | -0.232 | -0.016 |
|  | **<0.001** | **<0.001** | **<0.001** | **<0.001** | **<0.001** | **0.001** | 0.812 |
| Heart disease | 0.204 | 0.258 | 0.100 | 0.047 | 0.280 | -0.073 | 0.005 |
|  | **0.002** | **<0.001** | 0.139 | 0.498 | **<0.001** | 0.285 | 0.942 |

Note.

Bold indicate p < .05 uncorrected

ADHD = attention-deficit/hyperactivity disorder, ASD = autism spectrum disorder, ID = intellectual disability, NDCs = neurodevelopmental conditions (other NDCs includes e.g. communication disorders, specific learning disorders or motor disorders)

^a^ Measured with Social Responsiveness Scale-2 (SRS-2)

^b^ Measured with the Child Behavior Checklist (CBCL) or the Adult Behavior Checklist (ABCL)

^c^ Measured with Wechsler Intelligence Scales for Children or Adults-IV (WISC-IV/WAIS-IV)
