# Supplementary material for: Nonshared environmental factors in the aetiology of autism and other neurodevelopmental conditions: a monozygotic co-twin control study
Source: Mol Autism. 2022 Feb 19;13:8. doi: 10.1186/s13229-022-00487-5 (PMC8858556; doi:10.1186/s13229-022-00487-5)
Supplement: Supplementary file 4 — Additional file 4: Table S3. Results from within-pair linear regression analyses with birthweight difference in gram as main predictors of trait Neurodevelopmental conditions. [file 13229_2022_487_MOESM4_ESM.docx]

**Supplementary Table 3** Results from within-pair linear regression analyses with birthweight difference in gram as main predictors of trait Neurodevelopmental conditions

|  | ASD traits^a^  *b* (95% CI),  *p*-value, *Z*-score | ADHD traits^b^  *b* (95% CI),  *p*-value, *Z*-score | IQ^c^  *b* (95% CI),  *p*-value, *Z*-score |
| --- | --- | --- | --- |
| Birthweight discordance | -0.01625 (-0.02753, -0.00496), **0.005***, -2.82 | -0.00055 (-0.00082, -0.00028), **<0.0001***, -3.99 | 0.00574 (0.00036, 0.011129), **0.037***, 2.09 |

Note.

Bold indicate p < .05 uncorrected.

*Significant result after correcting for multiple comparisons using Benjamini-Hochberg procedure with false discovery rate (FDR) set at 5%.

ADHD = attention-deficit/hyperactivity disorder, ASD = autism spectrum disorder

^a^ Measured with Social Responsiveness Scale-2 (SRS-2)

^b^ Measured with the Child Behavior Checklist (CBCL) or the Adult Behavior Checklist (ABCL)

^c^ Measured with Wechsler Intelligence Scales for Children or Adults-IV (WISC-IV/WAIS-IV)
